# SUBMAXIMAL CONTROL AND EXPLOSIVE STRENGTH MAY BETTER DETECT COGNITIVE IMPAIRMENT THAN MAXIMAL STRENGTH

**DOI:** 10.1093/geroni/igae098.0509

**Published:** 2024-12-31

**Authors:** Ryan McGrath, Kelly Parker, Jeremy Hamm

**Affiliations:** North Dakota State University, Fargo, North Dakota, United States; North Dakota State University, Fargo, North Dakota, United States; North Dakota State University, Fargo, North Dakota, United States

## Abstract

Electronic handgrip dynamometry presents novel opportunities to feasibly collect muscle function attributes that are amenable to healthy behavior modification, which may have implications for identifying and delaying cognitive impairment. We sought to evaluate differences in electronic dynamometry derived aspects of muscle function by cognitive impairment status in older adults. Cognitive function was assessed with the Saint Louis University Mental Status exam, and standard scoring was used to categorize cognitive impairment. Participants squeezed an electronic handgrip dynamometer with maximal effort for determining strength capacity. Thereafter, participants attempted to squeeze the dynamometer at 25% of their maximal handgrip strength (HGS) for 10-seconds, and the coefficient of variation quantified submaximal control. Participants then squeezed the dynamometer as hard and fast as possible for calculating rate of force development (RFD): (delta force/delta time). The analytic sample included 125 adults aged ≥65-years (age: 70.83±4.79; 66.4% female). Submaximal control was significantly poorer in persons with a cognitive impairment (6.91±5.89) compared to those that were cognitively intact (4.80±3.42; p<0.05). Similar differences for submaximal control were observed in females with a cognitive impairment (7.97±6.70) compared to females without cognitive impairment (5.41±3.91; p<0.05). RFD was significantly lower in males with a cognitive impairment (51.0±25.7) relative to males without (72.94±26.51; p<0.05). No significant differences were observed for maximal HGS. Although several muscle indices are important for health, engaging in modifiable lifestyle behaviors, including physical activities that emphasize RFD and force control at different levels and durations, may serve as an intervention target for muscle and cognitive function.

